# MiR-1 downregulation correlates with poor survival in clear cell renal cell carcinoma where it interferes with cell cycle regulation and metastasis

**DOI:** 10.18632/oncotarget.3915

**Published:** 2015-04-23

**Authors:** Haibing Xiao, Jin Zeng, Heng Li, Ke Chen, Gan Yu, Junhui Hu, Kun Tang, Hui Zhou, Qihong Huang, Anping Li, Yi Li, Zhangqun Ye, Ji Wang, Hua Xu

**Affiliations:** ^1^ Department of Urology, Tongji Hospital, Tongji Medical College, Huazhong University of Science and Technology, Wuhan, China; ^2^ Institute of Urology, Tongji Hospital, Tongji Medical College, Huazhong University of Science and Technology, Wuhan, China; ^3^ The Wistar Institute, Philadelphia, PA, USA; ^4^ Department of Cell Death and Cancer Genetics, The Hormel Institute, University of Minnesota, Austin, MN, USA

**Keywords:** miR-1, ccRCC, proliferation, metastasis

## Abstract

MicroRNAs (miRNA) that are strongly implicated in carcinogenesis have recently reshaped our understanding of the role of noncoding RNAs. Here, we focused on the function and molecular mechanism of miR-1 and its potential clinical application in clear cell renal cell carcinoma (ccRCC). First, miR-1 was signiﬁcantly downregulated in 87.8% renal cancer samples compared with corresponding noncancerous tissues (NCT), which was significantly associated with clinical stage, T classification and poor overall survival. Functional study demonstrated that enforced overexpression of miR-1 in renal cancer cells inhibited proliferation and metastasis *in vitro* and *in vivo*. Conversely, miR-1 inhibitor silencing miR-1 expression promoted cell proliferation and metastasis in ccRCC. CDK4, CDK6, Caprin1 and Slug were each directly targeted for inhibition by miR-1 and restoring their expression reversed miR-1-mediated inhibition of cell cycle progression and metastasis. Taken together, our findings established a tumor suppressive role for miR-1 in the progression of ccRCC by targeting CDK4, CDK6, Caprin1 and Slug and suggested miR-1 can be served as a novel potential therapeutic target for ccRCC.

## INTRODUCTION

Renal cell carcinoma (RCC) accounting for nearly 3% of adult malignancies with about 65,150 new cases and 13,680 deaths estimated for 2013 in the United States [1]. Clear cell renal cell carcinoma is (ccRCC) the most common subtype of RCC and accounts for approximately 75–80% of these tumors with the highest rates of local invasion, metastasis, mortality and refractory to current treatments [2]. Apart from surgery, it is both chemotherapy and radiotherapy resistant. The present absence of biomarkers for early detection and follow-up of the disease is responsible for late diagnosis and subsequent poor prognosis. Hence, a better understanding of the mechanisms involved in the pathogenesis of ccRCC and more effective therapeutic approaches are urgently required.

MicroRNAs (miRNAs) are an abundant class of small non-coding RNAs that suppress gene expression at the post-transcriptional level by blocking mRNA translation or degrading target mRNAs [3]. Accumulating evidence has extended the function of miRNAs to both physiological and pathological conditions, including cancer [4]. A predominant and systemic alteration in miRNA expression during renal carcinogenesis has been indicated by present studies of miRNA expression profiling [5-9]. Among them, miR-1 is the most consistently decreased miRNA in renal cell carcinoma, suggesting the great potential miR-1 replacement therapy holds for cancer treatment. Through targeting multiple oncogenes and oncogenic pathways, miR-1 has been demonstrated to be a tumor suppressor gene that represses cancer cell proliferation and metastasis and promotes apoptosis by ectopic expression in lung cancer, colon cancer genitourinary cancer, head and neck cancer, thyroid cancer and hepatocellular cancer by targeting PIK3CA, MET, LASP1, TAGLN2, CCND2, FoxP1, HDAC4 and so on [10-15]. However, how miR-1 function in ccRCC pathogenesis remains largely unknown.

In the present work, we conﬁrmed that miR-1 expression was signiﬁcantly decreased in renal cancer tissues compared with the noncancerous tissues (NCT) in an expanded renal cancer cohort. We also first showed that miR-1 could lead to cell cycle arrest by directly targeting CDK4, CDK6 and Caprin1. What's more, Slug can be inhibited by miR-1. Moreover, miR-1 downregulation correlates with poor prognosis of ccRCC patients, suggesting that miR-1 might serve as a tumor-suppressor miRNA in the development and progression of ccRCC.

## RESULTS

### miR-1 downregulation correlated with ccRCC clinicopathologic characteristics and the overall survival of ccRCC patients

**Figure 1:**
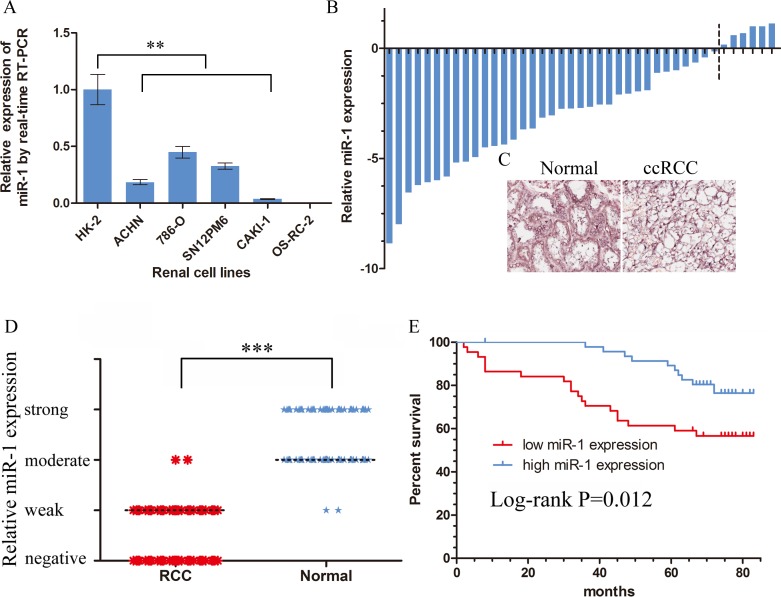
Downregulation of miR-1 in ccRCC correlated with poor patient survival Real-time PCR analysis of miR-1 expression in immortalized human renal tubule epithelial cell line HK-2 and indicated renal carcinoma cell lines. Data were plotted as the mean ± SEM of three independent experiments relative to HK-2 cells. **, *P* < 0.01. **B.** Relative expression of miR-1 in 41 pairs of ccRCC tumor tissues and their corresponding adjacent non-cancerous tissues (Δtumor-Δnormal). The average miR-1 expression was normalized by U6 expression. **C.** Expression of miR-1 in tumor tissues and their corresponding adjacent non-cancerous tissues by *in situ* hybridization (ISH). **D.** The expression level of miR-1 was measured by H-score. Negative (−, score: 0), weak (+, score: 1–4), moderate (++, score: 5–8) and strong (+++, score: 9–12). ***, *P* < 0.001. **E.** Kaplan-Meier analysis of correlation between the miR-1 level and overall survival of ccRCC patients with high (*n* = 47) and low (*n* = 43) miR-1 expression. In the Kaplan-Meier analysis, negative was recognized as low expression, weak and moderate were recognized as high expression.

To investigate the potential significance of miR-1 in the development and progression of ccRCC, we first examined the expression of miR-1 in clear cell renal cell carcinoma lines and tissues. Real-time PCR analysis demonstrated that miR-1 was ubiquitously expressed at lower levels in a panel of 5 human clear cell renal cell carcinoma lines than immortalized human proximal renal tubule epithelial cell line HK-2 (Figure 1A). In parallel, as showed in Figure 1B and Supplementary Figure S1, qRT-PCR showed that miR-1 was significantly downregulated in 87.8% (36/41) renal cancer tissues (*p* < 0.0001). These data strongly suggested that miR-1 expression was significantly suppressed in ccRCC.

The observed downregulated expression of miR-1 in renal cancer prompted us to further investigate the clinical relevance of miR-1 in the progression of ccRCC. To detect the expression patterns of miR-1 in the type of commercialized tissue microarrays, we employed *in situ* hybridization. The tissue microarrays contained 90 pairs of primary ccRCC specimens and their matched para-carcinoma tissue (Supplementary Table 1). The *in situ* hybridization analysis showed an overt reduction of miR-1 in the renal cancer specimens compared with adjacent non-cancerous tissues (Figure 1C, 1D). Furthermore, we did observe a significant difference in the distribution of the patients according to Clinical Stage (*P* = 0.013), T classification (*P* = 0.013) (Table 1). Kaplan-Meier analysis using the log-rank test was performed and the result demonstrated that patients with high miR-1 expression in their renal cancer had a longer median survival time than those with low miR-1 expression (Figure 1F). Taken together, these results suggested that miR-1 may play an important role in ccRCC progression.

**Table 1:** Patients characteristics and miR-1 expression of renal cell carcinoma from tissue microarray

		miR-1		P-value
		low	high	
Age(y)	>59	22	23	0.319
	<=59	22	23	
Gender	Male	24(47.1%)	27(52.9%)	0.691
	Female	20(51.3%)	19(48.7%)	
Clinical Stage	I	27(42.2%)	37(57.8%)	0.013
	II	9(50%)	9(50%)	
	III	6(100%)	0(0%)	
	IV	2(100%)	0(0%)	
T classification	T1	28(43.1%)	37(56.9%)	0.012
	T2	9(50%)	9(50%)	
	T3	7(100%)	0(0%)	

### miR-1 inhibited ccRCC cell proliferation and motility

**Figure 2:**
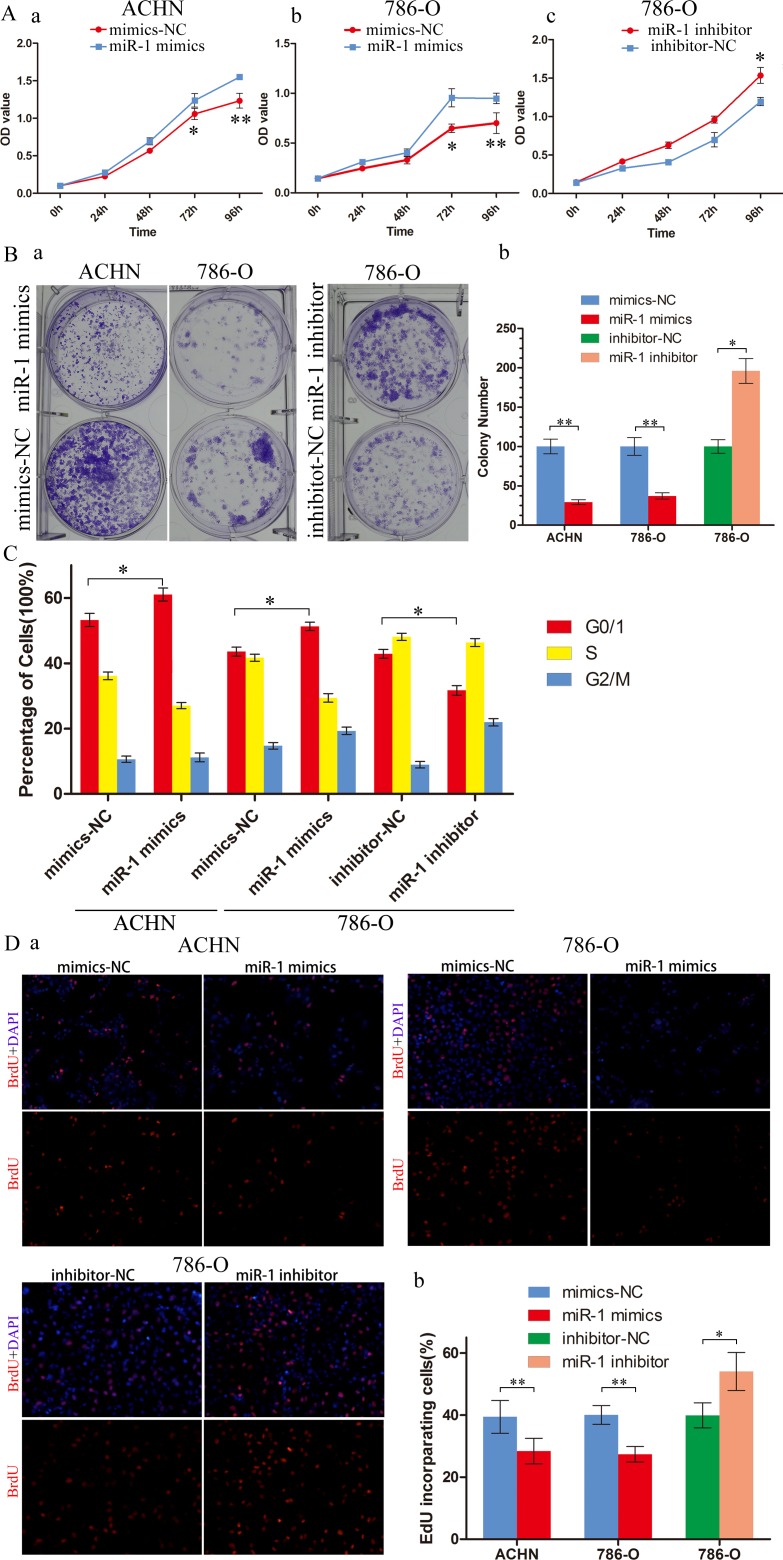
miR-1 attenuates ccRCC cell proliferation and motility ccRCC cells were transfected with 100 nM of indicated small RNA molecules. Results were plotted as the mean ± SEM of three independent experiments, with at least three replicates in each independent experiment. *, *P* < 0.05; **, *P* < 0.01. **A.** MTS assays revealed cell growth curves of indicated cells. **B.** Representative micrographs (left) and relative quantification (right) of crystal violet-stained cell colonies analyzed by clongenic formation. **C.** Flow cytometric determination of proportion of indicated cells in distinct cell cycle phases. **D.** Representative micrographs (left) and quantification (right) of EdU incorporated-cells in indicated engineered cell lines.

To explore the role of miR-1 in renal cancer cells, we transfected ACHN and 786-O with miR-1 mimics to upregulate miR-1 expression. After transfection with miR-1 mimics, a significant increase in miR-1 expression was confirmed using qRT-PCR (Supplementary Figure S2). MTS assay showed that the proliferation rate of ACHN and 786-O cells was significantly repressed after overexpression of miR-1 (Figure 2A); furthermore, the ability of colony formation was notably weakened (Figure 2B). To further dissect the biological events accompanying the alterations of cell proliferation caused by miR-1, FACS was applied to analyze changes of DNA content throughout various phases of the cell cycle. The result showed in Figure 2C, both ACHN and 786-O cells transfected with miR-1 displayed a significant increase in the percentages of cells in G1 phase. Furthermore, Edu incorporation assay confirmed that ACHN-miR-1 and 786-O-miR-1 contained less Edu-positive cells with newly synthesized DNA, 28.4% and 27.3%, respectively, than those in the control cell populations. To further understand the role of endogenous miR-1 in the modulation of cell proliferation, miR-1 inhibitors were used as antagonists to silence endogenous miR-1 expression (Supplementary Figure S3). We selected 786-O for further exploring, as for its relatively higher expression of miR-1 than other cancer cell lines. As showed in Figure 2A, 2B, 2C, 2D antagonizing miR-1 in 786-O drastically accelerated their proliferation as compared with their corresponding negative control cells in MTS, colony formation and Edu assay. Thus, our data suggested that miR-1 interfered with the G1-S transition of cell cycle progression and consequently abrogated the proliferation of renal cancer cells.

### miR-1 attenuates ccRCC cell migration and invasion

**Figure 3:**
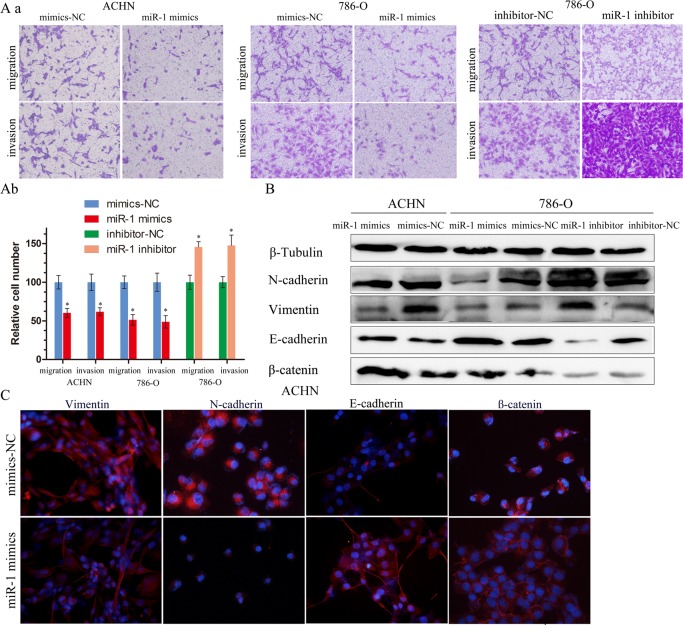
miR-1 attenuates ccRC cell migration and invasion **A. a.** Migration and invasion assay for renal cancer cells. Representative photographs were taken at ×200 magnification; number of migrated cells was quantified in ten random images from each treatment group. **b.** Results were the mean ± SEM from two independent experiments and plotted as percent (%) migrating cells relative to mimic-NC or inhibitor-NC. *P < 0.05. **B.** EMT-related proteins were determined by immunoblot analysis. β-Tubulin was used as loading control. **C.** Representative photographs of immunofluorescence were taken at ×200 magnification. ACHN cells were transfected with 100 nM of indicated small RNA molecules.

To determine whether miR-1 regulates ccRCC cell invasion and metastasis, we ﬁrst performed *in vitro* gain-of-function analyses by overexpressing miR-1 with miR-1 mimics in ACHN and 786-O cells. Migration and invasion assays were performed on the miR-1-infected cells. We found that ectopic expression of miR-1 signiﬁcantly suppressed the migration and invasion of ACHN and 786-O cells (Figure 3A). In contrast, the migration and invasion of 786-O cells increased when endogenous miR-1 was silenced with miR-1 specific inhibitors (Figure 3A). These observations suggest that miR-1 can suppress ccRCC cell migration and invasion *in vitro*. EMT played an important role in the process of migration and invasion, so we speculated that suppression of migration and invasion by miR-1 might impact EMT. To investigate this hypothesis, we examined the expression of the epithelial makers E-cadherin andβ-catenin, as well as the mesenchymal maker vimentin and N-cadherin. After infected with miR-1 in ACHN and 786-O cells, we found that E-cadherin and β-catenin expression dramatically increased with N-cadherin and vimentin expression reduced; whereas silencing miR-1 suppressed E-cadherin andβ-catenin expression, and induced vimentin in 786-O (Figure 3B). Immunoﬂuorescent staining also showed that miR-1 infection led to the upregulation of E-cadherin and the downregulation of vimentin and N-cadherin (Figure 3C). In addition, β-catenin was primarily located in the nucleus in ACHN cells; however, following miR-1 infection, β-catenin was absent from the nucleus, and instead, was localized at the plasma membrane. These results suggest that expression of miR-1 can reverse EMT in the renal cancer cell.

### miR-1 targeted cell cycle regulators CDK4, CDK6, Caprin1 and metastasis related gene Slug

**Figure 4:**
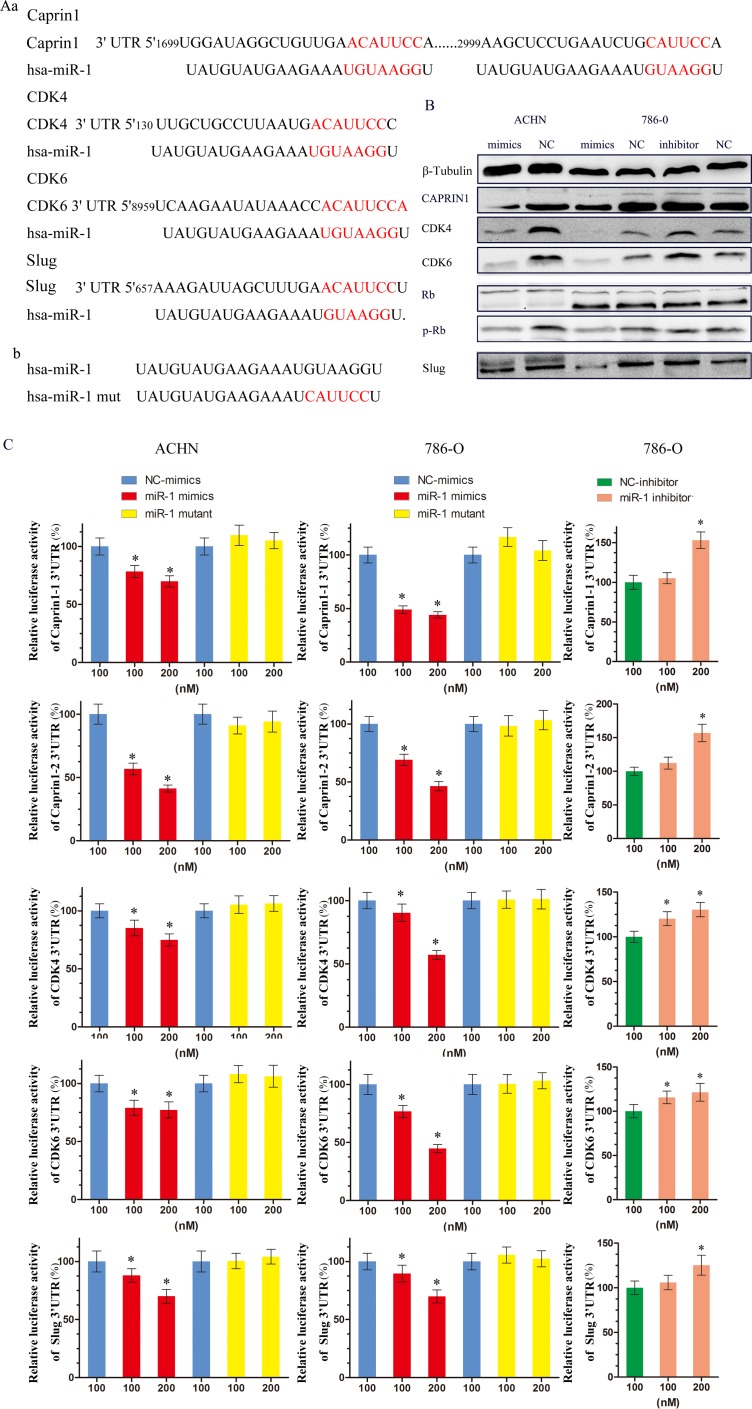
miR-1 targeted cell cycle regulators CDK4, CDK6, CAPRIN1 and metastasis related gene Slug **A. a.** Schematic miR-1 putative target sites in 3′ UTRs of CDK4, CDK6, Caprin1 and Slug. **b.** Sequence of miR-1-mut. **B.** WB analysis of the protein levels of CDK4, CDK6, Caprin1, slug, Rb and p-Rb in response to 100 nM of indicated small RNA molecules. **C.** Luciferase assay. Luciferase reporters harboring putative target sites in the 3′ UTRs of Caprin1 (Caprin1-1 and Caprin1-2), CDK4, CDK6 and Slug, were co-transfected with 100 and 200 nM of indicated small RNA molecules in ACHN and 786-O cells. Relative luciferase activity was plotted as the mean ± SEM of three independent experiments. *, *P* < 0.05.

To understand the underlying molecular mechanism by which miR-1 suppress ccRCC proliferation and metastasis, we searched for miR-1 targets using different computational methods, such as miRanda and TargetScan. Several of these possible target genes that have roles in cell proliferation and metastasis, including CCND1, CCND2, CDK4, CDK6, CDK9, Caprin1, Slug and so on. Since we have known cycle related genes CCND1, CCND2, CDK9 are reported the targets of miR-1 [16-19], we mainly focused on cell cycle related genes CDK4, CDK6, Caprin1 and metastasis related gene Slug. At first, two bioinformatics tools, TargetScan and miRanda, were used to further confirm that these genes were putatively potential targets of miR-1 (Figure 4Aa). Western blotting (WB) analysis consistently revealed that the expression level of 4 proteins were reduced in miR-1–overexpressing cells, whereas miR-1 inhibition elevated the levels of these proteins (Figure 4B). What's more, we also found that the levels of p-Rb were changed. At the same time, reporter assays showed that the activity of luciferase linked with the 3′UTR of CDK4, CDK6, CAPRIN1 or Slug was repressed in a dose-dependent manner in miR-1 mimics–transfected ACHN and 786-O cells, compared with those in control cells (Figure 4C). Conversely, inhibition of miR-1 caused a significant increase in luciferase reporter activities under the control of the 3′UTR of CDK4, CDK6, Caprin1 or Slug (Figure 4C). Of note, mutations brought into the seed sequence of miR-1 (Figure 4Ab) abolished its suppressive effects (Figure 4C). Collectively, these data suggest that miR-1 directly suppresses CDK4, CDK6, Caprin1 and Slug expression in clear cell renal cell carcinoma.

### Target genes partly suppresses functions initiated by miR-1

**Figure 5:**
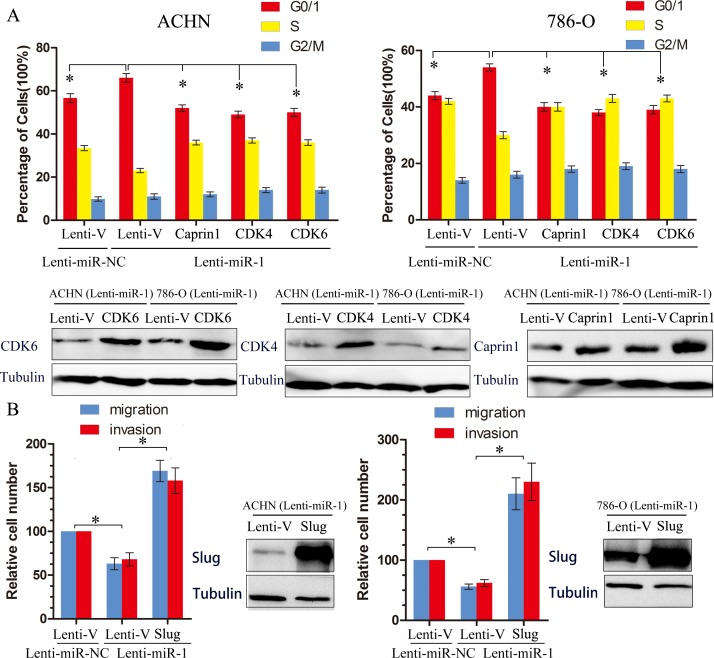
Target genes partly suppresses functions initiated by miR-1 **A.** The effect of ectopic restoration of Caprin1, CDK4 or CDK6 on proportions of indicated cells in distinct cell cycle phases as determined by flow cytometric analysis (top), the levels of Caprin1, CDK4 or CDK6 protein were measured by Western Blot (bottom). Lenti-V is the negative control for lentiviral-mediated CDK4, CDK6, Caprine1 and Slug ectopic overexpression. Lenti-miR-NC is the negative control for Lenti-miR-1. **B.** The effect of ectopic restoration of slug on proportions of indicated cells in distinct cell migration and invasion analysis. The levels of slug protein were measured by Western Blot (bottom). *, *p* < 0.05.

To clarify the functional significance of microRNA-mediated suppression of CDK4, CDK6 and Caprin1 in the induction of cell cycle arrest, we ectopically overexpressed these three genes in miR-1-overexpressing cells and assessed the cell cycle distribution of these by flow cytometry analysis. Restoration of CDK4, CDK6 and Caprin1 partially, but significantly, rescued the G1/S transition impaired by miR-1(Figure 5A), suggesting that downregulation of CDK4, CDK6, Caprin1 were functionally important for the inhibitory effect of miR-1 on ccRCC cell proliferation. Meanwhile, we found that expression of Slug partly abrogated migration and invasion initiated by miR-1 in renal cancer cells (Figure 5B). These results indicate that these genes serve as targets of miR-1, contributing to the effect of miR-1 on cell proliferation or metastasis.

### miR-1 inhibited ccRCC tumor growth in subcutaneously and orthotopic transplantation

**Figure 6:**
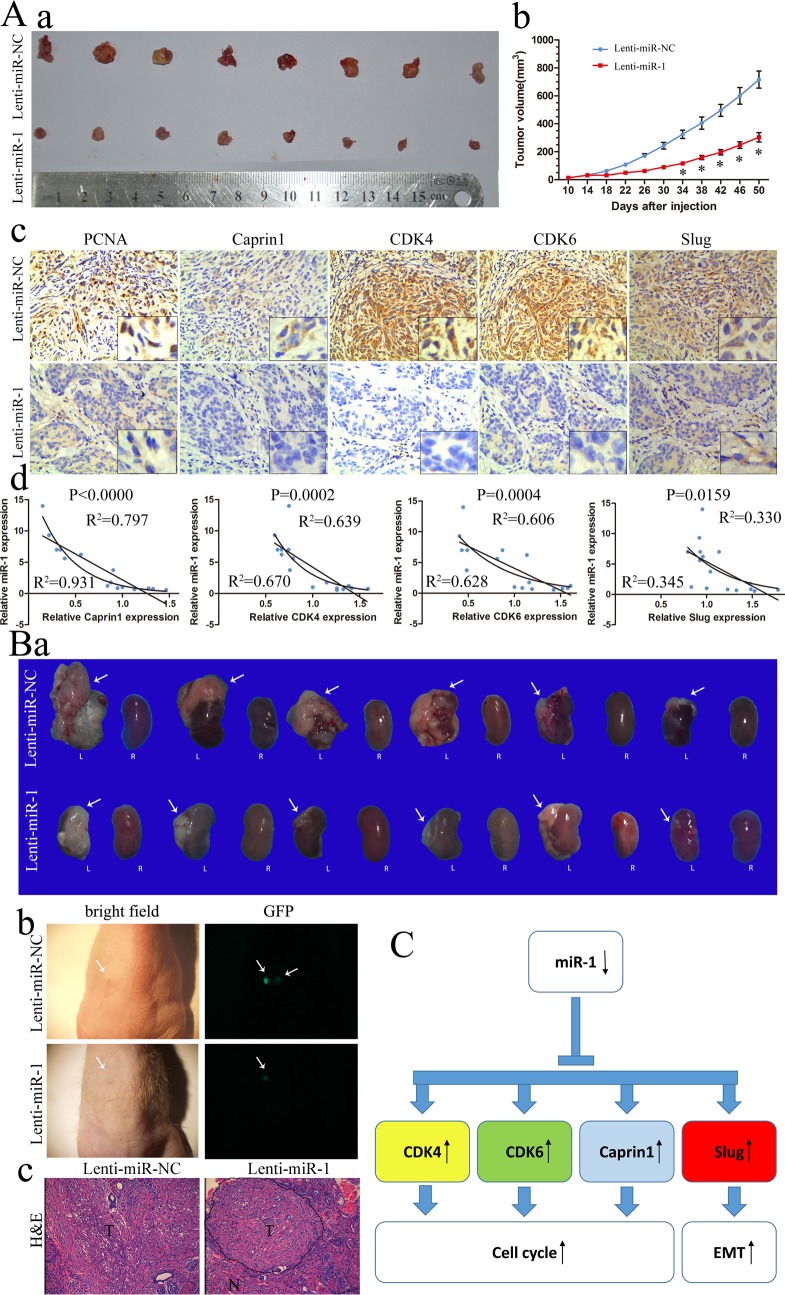
miR-1 inhibited ccRCC tumor growth in subcutaneously and orthotopic **A. a.** Photographs of tumors excised 51 days after inoculation of stably transfected cells into nude mice; **b.** mean tumor volume measured by caliper on the indicated days; **c.** IHC staining for PCNA, Caprin1, CDK4, CDK6 and Slug in slices of sectioned implanted tumors formed by indicated cells. Original magnification was ×200; **d.** Correlation of miR-1 with Caprin1, CDK4, CDK6 and Slug in subcutaneous transplantation tumor. **B. a.** Macroscopic appearance of the tumor xenograft (arrows) in nude mice from the 7th-week. L, left kidney; R, right kidney. **b.** Representative fluorescence images with primary tumors in the left kidney of nude mice after orthotopic injections of Lenti-miR-1 or Lenti-miR-NC for 7 weeks (arrows). **c.** H&E staining of the tumor xenograft. N, normal renal tissues; T, primary renal tumors. Original magnification was ×200. **C.** MiR-1 family modulates G1/S transition by regulating Cdk4/6-cyclin D complexes and Caprin1. Moreover, miR-1 can inhibit metastasis by downregulation of Slug.

Tumorigenicity assay was also carried out to confirm the tumor suppressive function of miR-1 *in vivo*. About 1 × 10^6^ ACHN cells infected with lenti-miR-1 or lenti-NC were injected subcutaneously into the axilla of nude mice (*n* = 8 per group). A significant increase in miR-1 expression was confirmed using qRT-PCR (Supplementary Figure S4). The Subcutaneous tumor formation assay was used to examine the proliferative ability of miR-1 overexpressed ACHN cells in nude mice. The results demonstrated lenti-miR-1 significantly reduced xenograft tumor growth (Figure 6Aa, 6Ab). In Figure 6Ac, subcutaneously transplation with high miR-1 expression, exhibited low levels of PCNA, CDK4, CDK6, Caprin1 and Slug respectively. In the fresh subcutaneous tumor, we detected the expression of miR-1 by RT-PCR and the expression of CDK4, CDK6, Caprin1 and Slug by Western Blot (Supplementary Figure S5). There are statistically correlations of the miR-1 level with the expression of CDK4, CDK6, Caprin1 and Slug (Figure 6Ad). We then further evaluated the relationships between the expression level of miR-1 and expression levels of CDK4, CDK6, Caprin1 or Slug in primary clear cell renal cell carcinoma samples, which were used in Figure 1. Consistent with subcutaneous transplantation tumors, expression level of miR-1 was also significantly correlated with expression levels of CDK4, CDK6 and Slug (*P* < 0.05) in patient samples (Supplementary Figure S6). While the relationship between miR-1 and Caprin1 in clinical samples was not as strong as it in xenografted tumors, which might be improved if more samples were involved in research. These results suggested a causal role for miR-1 in regulating these target genes *in vivo*.

There were reports have demonstrated that the renal orthotopic xenografts can develop primary renal tumors and give rise to metastases to multiple organs [20, 21]. To gain further insight into the effect of miR-1 on renal cancer, we established an orthotopic tumor model in nude mice with miR-1 and miR-NC infected ACHN cells. Tumor growth was surveilled by detecting GFP expression using the stereomicroscope. As shown in Figure 6Bb, fluorescence imaging showed a significant reduction of tumor growth in miR-1-overexpressed cells at 7th week. What's more, we found that the GFP signal may significantly strike renal fascia in miR-NC cells. Over a period of 7 weeks of transplant, there was an obvious decrease in tumor weight and size upon expression of miR-1 (*p* < 0.05) (Table 2 and Figure 6Ba). More specifically, tumors with miR-1 overexpression were universally telescoped and limited to kidney parenchyma, whereas tumors of control cells showed more aggressive growth. For example miR-NC tumors commonly infiltrated the kidney fascia, some even metastasized to various sites, including liver and cecum (Supplementary Figure S7). Although the *P* value of metastasis is 0.182 (Table 1), it seems that it may affect clear cell renal cell carcinoma metastasis if we had a large sample size.

These data further demonstrated that miR-1 functions as a vital tumor suppressor in ccRCC by suppressing tumorigenesis, local invasion and metastatic colonization.

**Table 2:** Incidence of renal tumor, tumor weight, invasion and metastasis in orthotopic xenografts

Group	No. of mice	Tumor incidence(%)	Tumor weight(mg) (mean ± SEM)	Beyond renal fascia rate(%)	Metastasis rate (%)
miR-1	6	6/6(100)	136.7±39.2	6/6(100)	3/6(50)
miR-NC	6	6/6(100)	49.3±21.2	1/6(16.7)	0/6(0)
P-value		NS^a^	0.000727^b^	0.015^c^	0.182^c^

a Not significant

b Tumor weight was calculated by subtracting the weight of the right kidney (normal) from the weight of the left kidney (implanted with tumor).

c P values were determined by Fisher's exact test.

## DISCUSSION

Identification of additional and essential molecular determinant(s) is impendent to designate alternative strategies to overcome resistance in ccRCC therapy [20]. It is well known that miRNAs are key components of tumorigenesis, as they participate in many cellular processes including cell proliferation, differentiation, and death. What's more, it may be an effective therapeutic strategies to tumor treatment [22-25]. We previously reported that miR-34a, which seems to be a cancer suppressor miRNA in renal cancer [26], and sensitized bladder cancer cells to cisplatin by directly targeting CD44 [27]. Recently, many papers have reported that a panel of miRNAs were altered in renal cancer tissues, suggesting that variations in the expression of miRNAs are common events in renal tumorigenesis, miR-1 has been reported to be one of the most significantly down-regulated in ccRCC tissues and cells [5, 6, 9]. In the present study, we investigated the biological role of miR-1 in human renal cancer. Our data present the demonstration that miR-1 is remarkably down-regulated in clear cell renal cell carcinoma lines and surgically excised ccRCC tumors. Particularly, miR-1 overexpression is significantly associated with improved survival in ccRCC patients. What's more, significant correlation was identified between miR-1 expression and ccRCC tumor stage and grade. In this context, we have first found that experimental restoration of miR-1 expression in ccRCC cells leads to suppression of Caprin1, CDK4 and CDK6, cell cycle arrest at G1/S checkpoint and disrupted proliferation of the cancer cells, whereas completely silencing miR-1 further upregulates Caprin1, CDK4 and CDK6 and promotes cell cycle progression. This is the first *in vivo* study on the functional characterization and mechanistic investigation of miR-1 in ccRCC.

Most microRNA have a dual role as either a tumor-promoting or -suppressive miRNA. However, miR-1 is frequently and consistently downregulated in various types of cancer and targeted multiple oncogenes and oncogenic pathways. MiR-1 was downregulated and acted as a tumor suppressor gene in hepatocellular carcinoma, colorectal cancer, prostate cancer, lung cancer, head and neck squamous cell carcinoma, rhabdomyosarcoma, bladder cancer and renal by targeting MET, FoxP1, HDAC4, Slug, PIK3CA, TAGLN2 *et al* [10-13, 28, 29]. Moreover, several lines of evidence indicate that miR-1 may perform novel functions in tumor proliferation by cell cycle arrest. Li L showed that transfection with miR-1 decreases the viability of rhabdomyosarcoma proliferation by targeting CCND2 [17]. The same results are proved by Leone V *etal* in thyroid cancer [18]. Zhang D have found that miR-1 suppresses G1/S phase transition by targeting CCND1 during the early stage of muscle regeneration. In this study, we have found miR-1 had a lower expression in clear cell renal cancer cell lines and tissues. Furthermore, loss of miR-1 expression is associated with poor prognosis in patients with renal clear cell carcinoma. We then found miR-1 inhibited clear cell renal cell carcinoma proliferation and induced G1/S cell cycle arrest. Gain of function assay showed that overexpression of miR-1 can suppress the expression of CCND1 and CCND2 (Supplementary Fig S8). We also found that experimental restoration of miR-1 expression in ccRCC cells leads to suppression of CDK4, CDK6 and Caprin1, cell cycle arrest at G1/S checkpoint and disrupted proliferation of the cancer cells, whereas completely silencing miR-1 further upregulated CDK4, CDK6 and Caprin1 and promotes cell cycle progression.

The cell cycle is regulated in part by cyclins and their associated serine/threonine cyclin-dependent kinases, or CDKs. CDK4, in conjunction with the D-type cyclins, mediates progression through the G1 phase when the cell prepares to initiate DNA synthesis [30]. Faussillon M have found CDK4 are frequent overexpression and had a specific correlation between relapse and CDK4 overexpression in Wilms' tumor [31]. CDK6, a cell cycle kinase which will also be activated when it binds D-type cyclins in early G1 phase. It has been found amplified or overexpressed in several malignancies including glioma, sarcoma, lymphoma and leukemia, is hyperactivated to promote cell proliferation and block differentiation during oncogenesis [32, 33]. Caprin-1 (cell cycle associated protein 1) have been showed that is needed for normal progression through the G1-S phase of the cell cycle by gene-targeting experiments [34], its levels are tightly correlated with cellular proliferation [35]. Further study had showed that down-regulation of caprin-1 by miR-16 has been considered as a mechanism that may contribute to the prolongation of the G1 phase of the cell cycle [36]. Our data has shown that CDK4, CDK6, Caprin1 can reverse miR-1 mediated cell cycle arrest.

It is very likely, therefore, that CDK4, CDK6 and Caprin1 might act cooperatively to initiate or promote tumor development and progression, and simultaneous silencing of all three genes might represent an effective and efficient strategy of suppressing oncogenesis because a cell that has lost the expression of one of them may still be able to proliferate [37]. As we all known, CDK4 plays a key role in mammalian development and cancer [37], but CDK4-null mutant mice are viable and cell proliferation is not significantly affected *in vitro* due to compensatory roles played by other CDKs(mainly CDK6) [37]. In other words, multiple targets regulated by an individual miRNA can act coordinately to regulate the same biological process more powerful [38]. CDK4/6-cyclin D complexes participate in the sequential hyper-phosphorylation of RB1 to repress RB inhibition of E2F, which positively regulates G1 to S-phase progression. So it will be very interesting that miR-1 had a powerful effect on leading to G0/G1 arrest by reducing multiple genes expression on the D-cyclin (ccnd1, ccnd2)–cdk4/6–INK4–Rb pathway (Figure 6C).

In addition to affecting proliferation, the role of miR-1 in metastasis is the other important aspect that must be considered in tumor-related research. We have found that miR-1 affected the renal cancer cell migration and metastasis *in vitro* and *in vivo.* In this study, overexpression of miR-1 greatly diminished N-cadherin and vimentin expression but increased the expression of E-cadherin andβ-catenin. Furthermore, miR-1 mediated E-cadherin induction led to the recruitment of β-catenin to the plasma membrane and inhibition of its nuclear translocation. Because β-catenin functions in a dual manner in epithelial cells, depending on its intracellular localization. A set of transcription factors that included SNAI1, SNAI2(SLUG), TWIST, and ZEB1/2 were initially identiﬁed as regulating epithelial-mesenchymal plasticity in embryonic morphogenesis and subsequently as suppressing CDH1 expression associated with various forms of EMT [39]. Our search to unravel the biological role of miR-1 in ccRCC metastasis identiﬁed Slug as a critical downstream target.

On the other hand, however, the mechanism via which miR-1 is downregulated in ccRCC remains uninvestigated. Investigators in previous studies identified several mechanisms for cellular regulation of these miRNA species, including epigenetic regulation (methylation and acetylation) of miR-1 in lung, prostate, and hepatocellular carcinoma [10, 40]. Anju Singh even found that NRF2-dependent epigenetic regulation of miR-1 reprograms glucose metabolism to promote cell proliferation and tumorigenesis through HDAC4. Taken together, it would be of great interest to further investigate whether miR-1 downregulation in ccRCC was attributable to genomic deletion and/or promoter methylation.

In summary, we combined clinical and experimental studies to determine the role of miR-1 in the progression of ccRCC. Our work provides new insights regarding the mechanisms of miR-1 activity in tumor growth and metastasis. Therefore, miR-1 may be a therapeutic target for the treatment of ccRCC.

## MATERIALS AND METHODS

### Human samples

Fresh-frozen samples. A total of 41 paired clear cell renal cell carcinoma and corresponding noncancerous tissues (NCT) were obtained sequentially from patients undergoing radical nephrectomy from the period of 2010–2014. Corresponding noncancerous tissues were acquired at least 5 cm away from the tumor site. Tissues specimens were snap frozen in liquid nitrogen before DNA and RNA extraction. The study protocol was approved by the ethics committee of Huazhong University of Science and Technology and Tongji Hospital and a written informed consent was obtained from all participants involved in this study.

Formalin-fixed, paraffin-embedded tissue specimens. In this study, we have used a tissue microarray (CHAOYING Biotechnology). Samples were obtained under informed consent from 90 patients with ccRCC who underwent operation from July 2006 to February 2008. Each tissues spot was accompanied with cases material including sex, age, pathologic type, pathologic grade, clinical stage and living condition.

Cell culture, infection, transfection ACHN, 786-O, SN12-PM6 and HK-2 cells were maintained in Dulbecco's modified Eagle's medium supplemented with 10% fetal bovine serum and 2 mmol/l l-glutamine in a humidified atmosphere of 5% CO2 maintained at 37°C. OS-RC-2 and CaKi-1 cells were cultured in RPMI-1640 supplemented with 10% fetal bovine serum and 2 mmol/l l-glutamine. All small RNA molecules were ordered from RiboBio (China), including miR-1 mimics, mimics negative controls (mimics-NC), miR-1 inhibitor and inhibitor negative controls (inhibitor-NC). MiR-1 mimics are double-stranded RNA molecules containing the miR-1 sequence, while miR-1 inhibitors are single-stranded RNA molecules containing miR-1 reverse complement sequence, which could competitively bind to endogenous miR-1. Cells were seeded into plate wells and incubated overnight, and then 100 nM of small RNA molecules were transfected into cells by using X-tremeGENE (Roche). For lentiviral-mediated ectopic overexpression of miR-1 target genes, the full-length cDNA of CDK4, CDK6, Caprin1 or Slug was cloned into pCDH-CMV-MCS-EF1-GreenPuro from SBI (CD513B-1) individually, and Lenti-V (pCDH-CMV-MCS-EF1-GreenPuro without any insert) served as a negative control. For lentiviral-mediated overexpression of miR-1, the miR-1 sequence (pri- miR-1) was cloned into H1-miRNA-CMV-GFP from GENECHEM, to generate the Lenti-miR-1 construct. MiR-NC (TTCTCCGAACGTGTCACGT) was cloned into the same backbone and the resultant construct Lenti-miR-NC served as a negative control. ACHN and 786-O cells were transducted with lentiviral particles at an MOI (Multiplicity of Infection) of 10 and 20 respectively, according to the manufacturer's instructions. To generate cell line with stable miRNA overexpression, ACHN cells were transducted with Lenti-miR-1 or Lenti-miR-NC and further verified with FACS.

### Immunofluorescence assay

ACHN cells were transfected with 100 nM of small RNA molecules and cultured on chamber slides for 48-72h, and then immunofluorescence assay was performed as previously described [41, 42] using E-cadherin, N-cadherin, β-catenin and Vimentin antibodies (1:100 dilution). Nuclei were stained with DAPI (10 mg/ml) for 10 min. Samples were examined with fluorescence microscope to analyze expression levels and subcellular localization of E-cadherin, N-cadherin, β-catenin and Vimentin.

### RNA extraction, quantitative real-time PCR (qRT-PCR) and semiquantitative reverse-transcription PCR (RT-PCR)

Total RNA of tissues and cells was extracted with TRIzol reagent (Invitrogen, Carlsbad, CA) according to the manufacturer's protocol with modification. Reverse transcription of microRNA and mRNA were done using RevertAid™ First Strand cDNA Synthesis Kit (Fermentas, Vilnius, Lithuania) and miProfile™ miRNA qPCR Primer (GeneCopoeia, Guangzhou, China). qRT-PCR analysis was performed with the Platinum SYBR Green qPCR Supermix UDG kit (Invitrogen, Carlsbad, CA) using synthesized primers from GeneCopoeia (Guangzhou, China).

### Cell viability, cell cycle, migratory and invasion assays

Cell viability was assessed at 0, 24, 48, 72 and 96 hours upon treatments by the 3-(4,5-dimethylthiazol-2-yl)-5-(3-carboxymethoxyphenyl)-2-(4-sulfophenyl)-2H-tetrazolium, inner salt (MTS) method (Sigma, USA) according to the manufacturer's instructions. The MTS have six replications. The colony formation have three replications. Fluorescence -activated cell-sorting (FACS) (BD, USA) analysis were done using propidium iodide (PI) stains for cell-cycle analysis according to the manufacturer's protocol with three replications. The 24-well transwell plate with 8 μm pore polycarbonate membrane inserts (Corning, New York, USA) was used to analyze the migration and invasive potential of cells according to manufacturer's protocol with three replications. For invasion assay, the membrane was coated with the matrigel (200 ng/ml) (BD Biosciences, Bedford, MA). For ACHN, the cell number is 1×10^5^ and migration/invasion time is 12 hours. For 786-O, the cell number is 3×10^4^ and migration/invasion time is 8 hours. After incubation, cells invading into the lower surface of the membrane insert were fixed in 100% methanol, stained with 0.05% crystal violet, and quantified by counting in 10 random fields as previously described [20].

### Xenograft subcutaneously and orthotopic implantations

Tumorigenicity in nude mice was determined as described previously [41]. Two groups of eight mice each were injected subcutaneously with prepared cells at the same site. Tumor onset was measured with calipers at the site of injection every 4-5 days by two trained laboratory staffs at different times on the same day 10 days after injection when appreciable tumor formed subcutaneously. Tumor volume was calculated using the formula, V = 0.5ab^2^, where a represents the larger and b represents the smaller of the two perpendicular indexes. Animals were killed 50 days after injection and tumors were weighed. For *in vivo* orthotopic xenograft studies, 1 × 10^6^ ACHN cells stably expressing miR-1 or miR-NC were injected into the left kidney of male BALB/c nude mice at 4-5 weeks of age as previously described [20]. 7 weeks after the implantation of the xenografts, animals were euthanized and xenografts were harvested, and assessed for tumor weight, local invasion and distant metastasis. Renal tumors from xenografts were flash frozen in liquid nitrogen and stored at −80°C for RNA extraction. Formalin-fixed, paraffin-embedded ccRCC xenografts were assessed by hematoxylin and eosin (HE) staining and evaluated for target gene expression. Nude mice were manipulated and cared according to NIH Animal Care and Use Committee guidelines in the Experiment Animal Center of the Tongji Medical College of Huazhong University of Science and Technology, China.

### Luciferase assays

Briefly, ACHN and 786-O cells were seeded in 96-well plates (5000 cells per well) and co-transfected with 100 ng psicheck2 Luciferase vector containing target genes 3′UTR with 100 nM or 200 nM miR-1 mimics or mutant mimics or NC. Forty-eight hours after transfection, Dual- Luciferase Reporter Assay (Promega) was performed according to the manufacturer's instructions, as previously described [43].

### Immunohistochemistry and *in situ* hybridization

Immunohistochemical staining was performed as previously described, using CDK4 (Abcam), CDK6 (Abcam), Caprin1 (Gene Tex), PCNA (Epitomics), Slug (Abcam) antibody. *In situ* hybridization was performed using a miR-1 probe from Exiqon (miRCURY LNA detection probe 5′and3′-DIG (digoxigenin)-labeled). The probe was detected using digoxigenin antibody (Abcam), LSAB2 System-HRP (Dako Denmark A/S, Glostrup, Denmark) and liquid DAB+ Substrate Chromogen System (Dako) according to the manufacturer's instructions. The results of immunostaining and hybridization were independently scored by two pathologists from Tongji Hosptial in a blind manner. The scoring was based on the intensity and extent of staining and was evaluated according to the following histological scoring method. The mean proportion of staining cells per specimen was determined semi-quantitatively and scored as follows: 0 for staining <1%, 1 for 1–25%, 2 for 26–50%, 3 for 51–75%, and 4 for >75% of the examined cells. Staining intensity was graded as follows: 0, negative staining; 1, weak staining; 2, moderate staining; 3, strong staining. The histological score (H-score) for each specimen was computed by the formula: H-score=Proportion score*Intensity score. A total score of 0–12 was calculated and graded as negative (−, score: 0), weak (+, score: 1–4), moderate (++, score: 5–8) or strong (+++, score: 9–12) [44]. In the Kaplan-Meier analysis, negative was recognized as low expression, weak and moderate were recognized as high expression.

### Statistical analysis

All statistical analyses were carried out using SPSS 18.0 statistical software. The Kaplan-Meier method was used to establish survival curves, and the survival differences were compared using the log-rank test. Continuous data were compared using Student's 2-tailed *t* test. Data are represented as mean ±SEM. In all cases, *P* < 0.05 was considered statistically significant. *, *p* < 0.05; **, *p* < 0.01; ***, *p* < 0.001.

## SUPPLEMENTARY MATERIALS, FIGURES, TABLES


