# Correction: MiR-1 downregulation correlates with poor survival in clear cell renal cell carcinoma where it interferes with cell cycle regulation and metastasis

**DOI:** 10.18632/oncotarget.27357

**Published:** 2019-12-24

**Authors:** Haibing Xiao, Jin Zeng, Heng Li, Ke Chen, Gan Yu, Junhui Hu, Kun Tang, Hui Zhou, Qihong Huang, Anping Li, Yi Li, Zhangqun Ye, Ji Wang, Hua Xu

**Affiliations:** ^1^ Department of Urology, Tongji Hospital, Tongji Medical College, Huazhong University of Science and Technology, Wuhan, China; ^2^ Institute of Urology, Tongji Hospital, Tongji Medical College, Huazhong University of Science and Technology, Wuhan, China; ^3^ The Wistar Institute, Philadelphia, PA, USA; ^4^ Department of Cell Death and Cancer Genetics, The Hormel Institute, University of Minnesota, Austin, MN, USA; ^*^ Haibing Xiao and Jin Zeng contributed equally to this work

**This article has been corrected:** Due to errors in image selection, two small pictures were partially overlapped in Figure 3A - the mimics-NC are the same as the inhibitor-NC in the migration group of 786-O. The corrected Figure 3 is shown below. The authors declare that these corrections do not change the results or conclusions of this paper.

Original article: Oncotarget. 2015; 6:13201–13215. 13201-13215. https://doi.org/10.18632/oncotarget.3915

**Figure 3:**
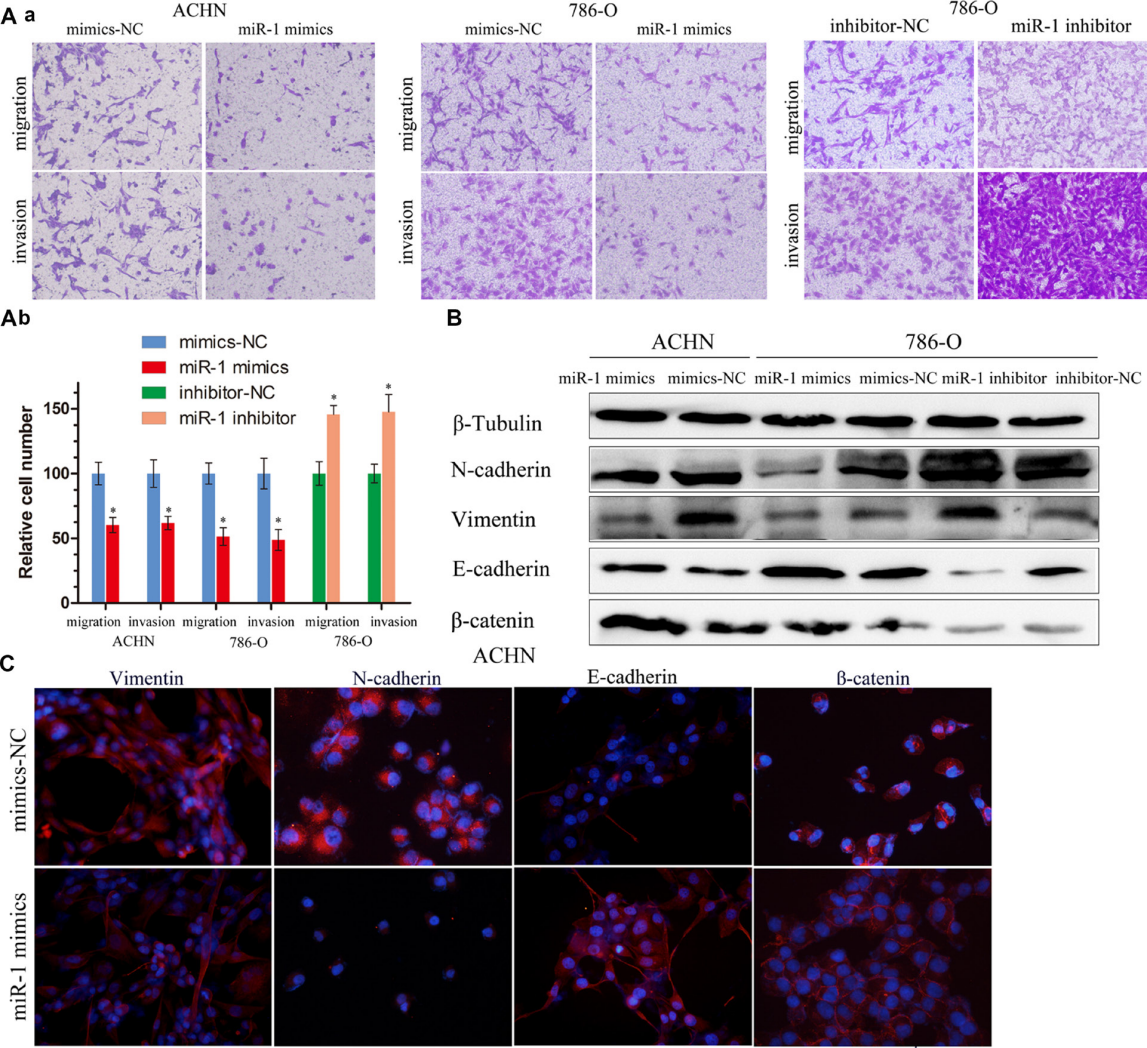
miR-1 attenuates ccRC cell migration and invasion.

(**A**. **a**) Migration and invasion assay for renal cancer cells. Representative photographs were taken at ×200 magnification; number of migrated cells was quantified in ten random images from each treatment group. (**b**) Results were the mean ± SEM from two independent experiments and plotted as percent (%) migrating cells relative to mimic-NC or inhibitor-NC. *P < 0.05. (**B**) EMT-related proteins were determined by immunoblot analysis. β-Tubulin was used as loading control. (**C**) Representative photographs of immunofluorescence were taken at ×200 magnification. ACHN cells were transfected with 100 nM of indicated small RNA molecules.

