# Supplementary material for: MiR-1 downregulation correlates with poor survival in clear cell renal cell carcinoma where it interferes with cell cycle regulation and metastasis
Source: Oncotarget. 2015 Apr 23;6(15):13201–15. doi: 10.18632/oncotarget.3915 (PMC4537008; doi:10.18632/oncotarget.3915)
Supplement: Supplementary file 1 [file oncotarget-06-13201-s001.pdf]

MiR-1 downregulation correlates with poor survival in clear cell renal cell carcinoma where it interferes with cell cycle regulation and metastasis

Supplementary Material

Fig S1

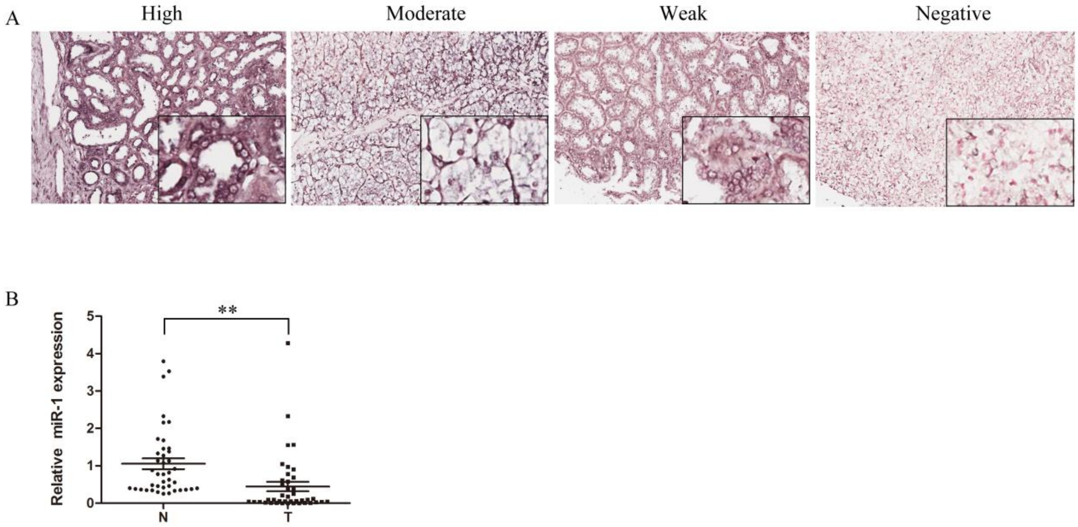

Figure S1: A. The representative images for *in situ* hybridization staining of miR-1 in different categories (negative, weak, moderate and high).

B. Relative expression of miR-1 in 41 pairs of ccRCC tumor tissues and their corresponding adjacent non-cancerous tissues (Δtumor-Δnormal). The average miR-1 expression was normalized by U6 expression. ** P<0.01

Fig. S2

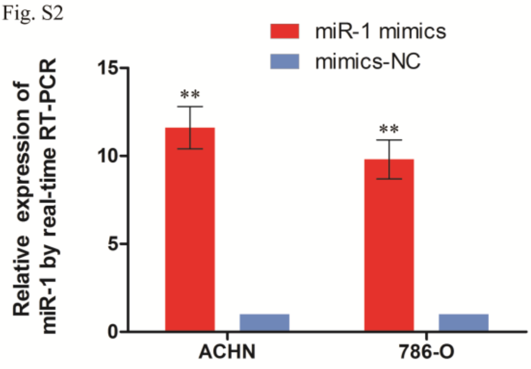

Figure S2: Real-time PCR analysis of miR-1 expression in ACHN and 786-O cells transfected with negative control or miR-1 mimics. Transcript levels were normalized by U6 expression. Error bars represent the means of three independent experiments. **, P<0.01.

Fig. S3

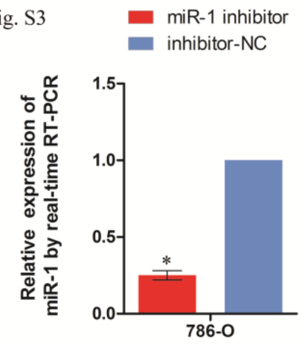

Figure S3: Real-time PCR analysis of miR-1 expression in 786-O cells transduced with negative control or miR-1 inhibitor. Transcript levels were normalized by U6 expression. Error bars represent the means of three independent experiments. *, $P<0.05$.

Fig. S4

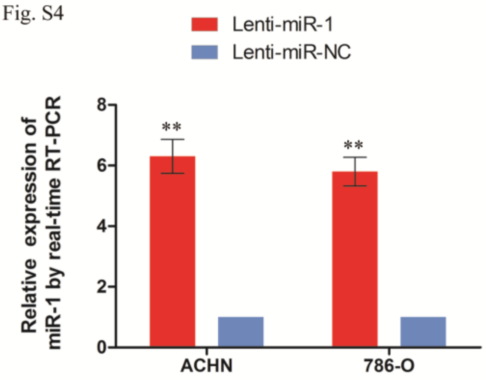

Figure S4: Real-time PCR analysis of miR-1 expression in ACHN and 786-O cells transduced with Lenti-miR-1 or Lenti-miR-NC. Transcript levels were normalized by U6 expression. Error bars represent the means of three independent experiments. **, P<0.01.

Fig. S5

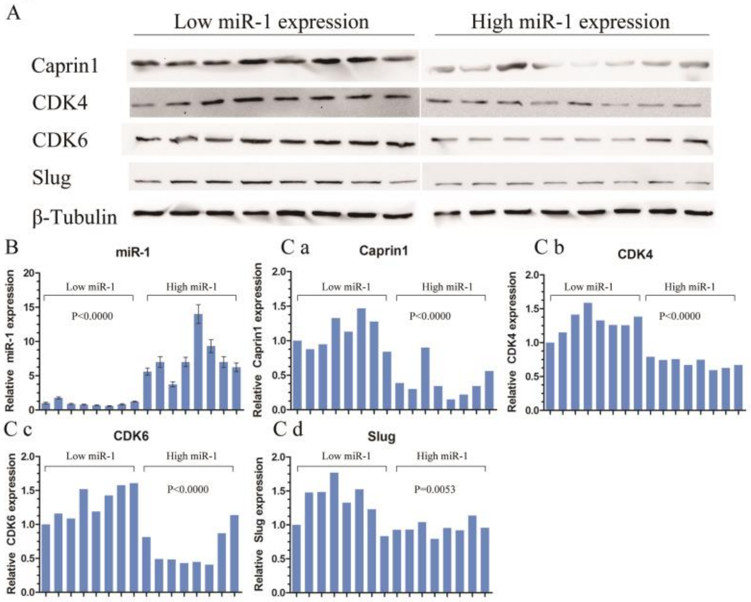

Figure S5: A. WB analysis of the protein levels of Caprin1, CDK4, CDK6, and Slug in subcutaneous transplantation tumor. β-Tubulin was used as a loading control.

B. miR-1 was measured by qRT-PCR in subcutaneous transplantation tumor. U6 as a control.

C. Protein levels were quantified by scanning densitometry and scored.

Fig S6

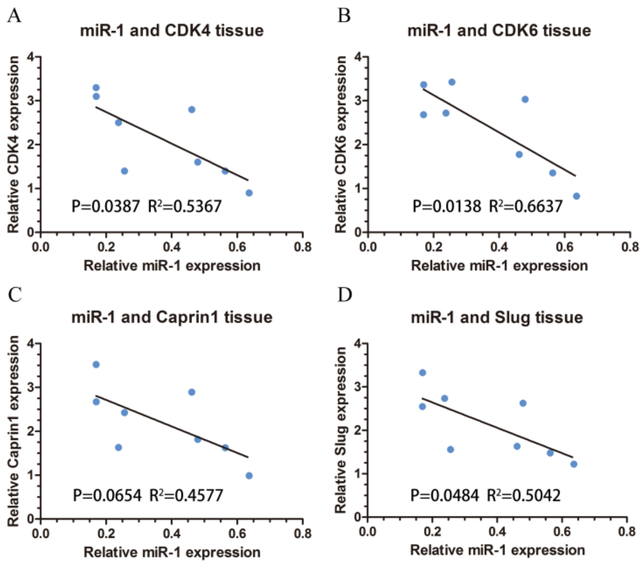

Figure S6: Correlation of miR-1 with Caprin1, CDK4, CDK6 and Slug in primary clear cell renal cell carcinoma samples.

Fig. S7

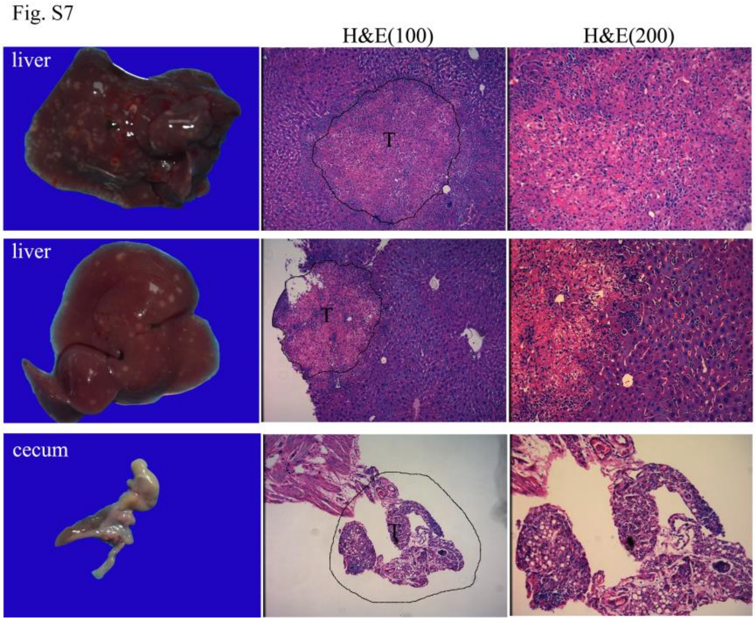

Figure S7: ACHN cells stably overexpressing Lenti-miR-NC metastasized to multiple organs and H&E dye (×100 and ×200).

Fig. S8

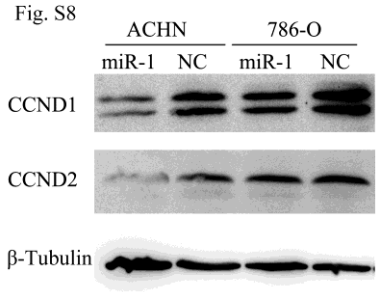

Figure S8: WB analysis of the protein levels of CCND1 and CCND2 in response to transfected with miR-1 mimics or miR-NC.

Table S1

		No of case (%)
Age(y)	>59	45(50)
	<=59	45(50)
Gender	Male	51(56.7)
	Female	39(43.3)
Clinical Stage	I	64(71.1)
	II	18(20.0)
	III	6(6.7)
	IV	2(2.2)
T classification	T1	65(72.2)
	T2	18(20)
	T3	7(7.8)
